# Super-resolution mapping of anisotropic tissue structure with diffusion MRI and deep learning

**DOI:** 10.1038/s41598-025-90972-7

**Published:** 2025-02-24

**Authors:** Alfredo Ordinola, David Abramian, Magnus Herberthson, Anders Eklund, Evren Özarslan

**Affiliations:** 1 Department of Biomedical Engineering, Linköping University, Linköping, Sweden; 2 Center for Medical Image Science and Visualization, Linköping University, Linköping, Sweden; 3 Department of Mathematics, Linköping University, Linköping, Sweden; 4 Department of Computer and Information Science, Linköping University, Linköping, Sweden

**Keywords:** Brain, Machine learning, Magnetic resonance imaging

## Abstract

Diffusion magnetic resonance imaging (diffusion MRI) is widely employed to probe the diffusive motion of water molecules within the tissue. Numerous diseases and processes affecting the central nervous system can be detected and monitored via diffusion MRI thanks to its sensitivity to microstructural alterations in tissue. The latter has prompted interest in quantitative mapping of the microstructural parameters, such as the fiber orientation distribution function (fODF), which is instrumental for noninvasively mapping the underlying axonal fiber tracts in white matter through a procedure known as tractography. However, such applications demand repeated acquisitions of MRI volumes with varied experimental parameters demanding long acquisition times and/or limited spatial resolution. In this work, we present a deep-learning-based approach for increasing the spatial resolution of diffusion MRI data in the form of fODFs obtained through constrained spherical deconvolution. The proposed approach is evaluated on high quality data from the Human Connectome Project, and is shown to generate upsampled results with a greater correspondence to ground truth high-resolution data than can be achieved with ordinary spline interpolation methods. Furthermore, we employ a measure based on the earth mover’s distance to assess the accuracy of the upsampled fODFs. At low signal-to-noise ratios, our super-resolution method provides more accurate estimates of the fODF compared to data collected with 8 times smaller voxel volume.

## Introduction

Diffusion magnetic resonance imaging (diffusion MRI) is an MRI modality used to probe and characterize the diffusion process of water molecules within the tissue. Diffusion MRI has proven sensitivity to many diseases affecting the tissue, making it an indispensable tool in diagnostic medicine^1,2^. Diffusion of molecules is influenced by the ambient environment, which imprints its signature on the diffusion MRI signal. Of particular interest to us are the microstructural characteristics of the tissue that influence the diffusion MRI signal, which could be instrumental in not only improving the diagnostic utility of MRI, but also address rather fundamental questions regarding the make up and organization of the tissues^3^.

For example, one of diffusion MRI’s principal uses involves estimating the local orientation of axonal fibers within white matter in the brain. These, in turn, can be used to estimate the long-range connections inside the brain, through a procedure known as *tractography*^4–6^, which consists of alternately sampling the local fiber orientation at a given position in the brain and taking a small step in that direction. Performing this procedure starting from multiple points within the brain makes it possible to reconstruct the pathways of the axonal bundles inside the brain.

There are multiple ways of describing and summarizing the local diffusion properties of water molecules, which differ in the amount of precision they afford, the complexity of the acquisition process required to estimate them, and their suitability for various purposes. Diffusion tensor imaging (DTI)^7^ is the most common such method, in which a tensor is fitted to the diffusion signal at every brain voxel. For voxels containing a single fiber population, this provides a suitable estimation of its primary orientation, but the method is incapable of properly representing diffusion along multiple crossing fiber populations.

To address this challenge, a number of higher order models have been proposed over the years^8–18^. Among these, the constrained spherical deconvolution (CSD) method^15^ has been widely employed in tractography studies. In this approach, the signal is envisioned to arise from a continuous distribution of fibers. The associated density function is referred to as the “fiber orientation distribution function (fODF),” which is estimated by deconvolving the detected signal with a response function taken to be the same for all fibers. Such models rely on diffusion MRI data with a relatively large number of diffusion-weighted MRI acquisitions each having a measurement sensitized to diffusion taking place along a different orientation^19^. Moreover, the data are to be acquired preferably at a larger diffusion-weighting, resulting in a reduced signal-to-noise ratio (SNR) compared to what can be achieved in DTI acquisitions. In order to address these issues, spatial resolution is typically reduced. Having voxels of size 2–3 mm is thus common in conventional diffusion MRI acquisitions. However, tractography has been shown to significantly benefit from both high angular and spatial resolutions^20^, both of which are often not achievable in clinical practice at the same time due to long scan times.

To overcome this serious limitation, we introduce a deep-learning-based super-resolution technique that provides an accurate upsampling of data collected for CSD analysis, thus providing fODF-valued images with voxels considerably smaller than the resolution employed in data acquisition. Furthermore, relying on such super-resolution technique would enable the use of low-resolution data, which inherently feature a lower noise content when compared to high-resolution data, in various analyses.

Adaptation of deep learning for achieving super-resolution has been a popular research topic during recent years. Deep learning methods have already been incorporated into clinical acquisitions for generating high-resolution reconstructions of images accounting for space-dependent noise patterns^21^. Most of the work has focused on two-dimensional (2D) images^22–24^, and much less work has extended the methods from 2D to 3D^25–28^. However, diffusion MRI data is 4D, as many volumes (e.g., 7–300) are collected with diffusion weighting along different directions and with different b-values. Unfortunately, existing deep learning frameworks do not support 4D convolutional neural networks (CNNs). Since the order of the collected volumes is not important, compared to functional MRI which has a time dimension, it is sufficient to use a multi-channel 3D CNN. However, doing so would require too much GPU memory to work on the full dataset. To use super-resolution techniques for diffusion MRI data can therefore be done in different ways; independently for a small number of adjacent 2D slices, independently for a small number of 3D volumes, or independently for all measurements within a small neighborhood of each voxel. While the first two approaches can use a larger spatial context, they will run into GPU memory problems when using many measurements at the same time.

In the context of diffusion MRI data, previous works aim to improve the angular resolution^29^, spatial resolution^30,31^, a combination of both^32^, or quality^33,34^ of this type of data. For example, Qin et al.^30^ used deep learning to obtain super-resolved diffusion MRI data. The input in their framework involved all measurements for a small number of voxels. There are a number of differences compared to our work. First, they apply the super-resolution to raw diffusion measurements, or more precisely a learned sparse dictionary, while we apply it to 45 spherical harmonic (SH) coefficients in a spherical harmonics (SH) representation of the fODFs obtained through CSD. Second, their network generates high-resolution maps of scalar parameters representing some microstructural features (orientation dispersion, intra-cellular volume fraction and cerebrospinal volume fraction) while we generate a high-resolution version of the 45 SH coefficients. Our work is thereby more aimed at tractography. Luo et al.^35^ instead used a generative adversarial network (GAN) architecture to directly upsample low-resolution diffusion MRI data to a higher resolution. Although a 3D CNN was used inside the GAN, each sub-volume contained only 11 $\times$ 11 $\times$ 11 voxels to be able to fit all 95 measurements at the same time.

## Methods

All data processing was done using custom Python (version 3.9) scripts and Jupyter notebooks. All processing of diffusion MRI data was done using the dipy toolbox^36^ (version 1.5) for Python. Deep learning models were implemented in TensorFlow (version 2.4.1)^37^.

### Data

Data used for all analyses were obtained from the publicly-available WU-Minn Human Connectome Project (HCP)^38^ database. We used the 100 unrelated adult subject sub-group (54% female, mean age = 29.11 years, range = 22–36). Five of the subjects were excluded due to incomplete WM coverage of the diffusion MRI data, leaving a total of 95 subjects. The data have been previously subjected to a minimal preprocessing pipeline^39^. The HCP diffusion MRI data have isotropic voxels of size 1.25 mm, and consist of 288 diffusion-weighted volumes, with 3 shells of data with b-values of 1000, 2000 and 3000 s/$\hbox{mm}^2$ and 90 diffusion directions each, in addition to 18 volumes collected with no diffusion weighting. Besides these 18 volumes, only the volumes in the 3000 b-value shell were used, as the fitting was done with a single-shell model, and constrained spherical deconvolution (CSD) benefits from a strong diffusion encoding. It should be noted that the acquisition time of the subset of data employed in this study is approximately 18.3 min^38^.

In order to simulate low-resolution data, the original diffusion MRI volumes, featuring a voxel size of $1.25 \times 1.25 \times 1.25\,\hbox{mm}^3$, were downsampled by averaging the signal in each non-overlapping $2 \times 2 \times 2$ voxel region, yielding “low-res” images having voxels of volume $2.5 \times 2.5 \times 2.5\,\hbox{mm}^3$. Given that in MRI acquisition the signal within a voxel integrates the contributions from all components within the voxel, such a downsampling approach mimics the effects of having acquired MRI data with half the spatial resolution in each axis.

The HCP data include a parcellation volume for each subject, where each brain voxel is classified as belonging to one of a number of anatomical regions and tissue types. These tissue labels were used in order to evaluate the performance of the upsampling methods on different tissue types, namely white matter (WM), gray matter (GM) and cerebrospinal fluid (CSF).

### Data processing

For every subject, fODFs were estimated at every voxel by fitting a constrained spherical deconvolution (CSD) model^15^. The WM response function used for deconvolution, representing the expected measured diffusion signal for a single fiber bundle, was estimated from highly anisotropic voxels in and near the corpus callosum. The fODFs were transformed into the spherical harmonic (SH) domain, which constitutes a natural representation for spherical functions. The SH basis used was the one employed by Descoteaux et al.^40^ in their work on Q-ball imaging, with a maximum order of 8, resulting in 45 real SH coefficients per voxel. The use of the SH domain has several advantages over using raw data or the fODFs spherical functions directly. First, it allows a full description of the spherical functions using a limited number of coefficients. Furthermore, it provides a generic domain independent from the specific sampling scheme chosen for the fODFs, and which can likewise be calculated from any chosen spherical sampling. It is therefore possible, at least in theory, to use our trained super-resolution network on diffusion data collected with another sampling scheme.

Due to the differential proton-density and magnetic relaxation effects over tissue types, fODFs generated in this way do not correspond to true probability distributions, i.e., their integral over the surface of the sphere is not equal to unity. In order to obtain true probability distributions, the SH coefficients at each voxel were normalized by dividing them by $\sqrt{4 \pi } A_0$, where $A_0$ represents the 0-th SH coefficient. This has the added advantage of requiring one fewer SH coefficient, as $A_0$ becomes $1/\sqrt{4\pi }$ for all voxels.

The described procedure was carried out separately on the low-resolution and high-resolution diffusion MRI data.

### Neural network model

We used a fully-connected neural network model to upsample the low-resolution SH data to its original resolution. The network was provided with SH coefficients from a cubic region of the low-resolution data as input, and was trained to output the SH coefficients of the 8 high-resolution voxels corresponding to the central voxel of the low-resolution input region. Specifically, an array of 1188 numbers ($3 \times 3 \times 3$ voxels with 44 SH coefficients per voxel, flattened to a vector) were provided to the network which yielded 352 numbers which were subsequently mapped to an array representing $2 \times 2 \times 2$ voxels with 44 SH coefficients per voxel.

**Fig. 1:**
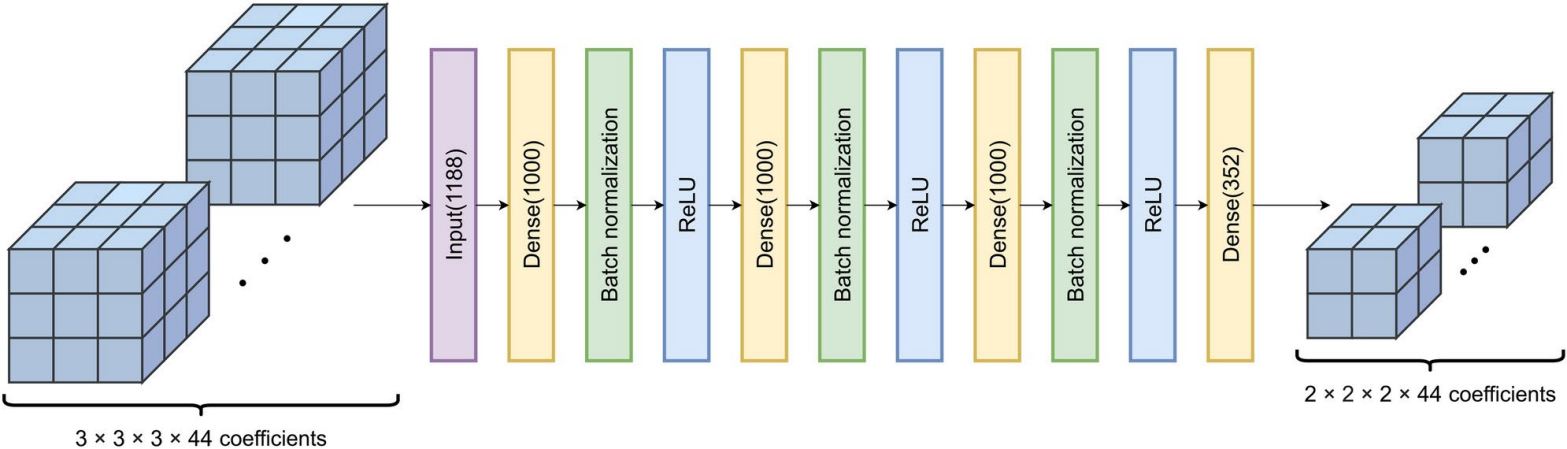
Schematic representation of super-resolution network. The 44 SH coefficients for each voxel in a region comprising $3 \times 3 \times 3$ voxels of low-resolution data are flattened into a vector and provided as input. The network consists of three blocks, each comprising a dense layer with 1000 nodes, a batch normalization layer, and a ReLU activation. The output is produced by a final dense layer with 352 nodes, which can be rearranged into the 44 SH coefficients of each voxel in the $2 \times 2 \times 2$ voxel region corresponding to an upsampled version of the central voxel of the input region.

The neural network consisted of several fully-connected layers, with batch normalization and ReLU activation functions for the hidden layers, and no activation function for the final layer. The network was trained with mean squared error loss, Adam optimizer^41^, and a batch size of 512 for 100 epochs. A grid search was used to select the number of fully-connected layers and the number of nodes per layers. It was found that 3 layers of 1000 nodes each resulted in a good trade-off between validation performance and degree of overfitting. Figure 1 presents a schematic representation of the employed network. SH data from 50 subjects were used for training, with 10 subjects used for validation, and the remaining 35 for testing. Data were split on the subject level, to avoid data leakage^42^. As every low-resolution brain voxel constitutes a datapoint, and with an average of 130 000 low-resolution voxels per subject, this resulted in 6.7 million, 1.4 million, and 4.7 million training, validation, and test data points, respectively. The final network has a total of 3.5 million trainable parameters, and the training took approximately 90 min. on an Nvidia Quadro RTX 8000 GPU.

It should be noted that CNNs would be well suited for this upsampling task, due to their efficient use of parameters and the large spatial context that is considered for each predicted output. However, an equivalent CNN implementation to the proposed fully-connected network would require 3-dimensional filters and 44 input channels, which would place very substantial memory requirements on the GPU (unless the CNN works on subvolumes^35^). By comparison, the proposed network has minimal GPU requirements which are fulfilled by most consumer-grade GPUs in the market. Nevertheless, the necessity of providing spatial context in the network input was recognized. We experimented with input regions of size $3 \times 3 \times 3$ and $5 \times 5 \times 5$. However, the larger input region did not provide improved results over the smaller, as the substantial increase in the number of network parameters (from 3.5 to 7.8 million) led to increased overfitting. Henceforth, all results presented are for a network with an input size of $3 \times 3 \times 3$.

### Interpolation

For the purposes of comparison, the low-resolution data were also upsampled using 3D spline interpolation of orders from 0 to 5, where orders 0 and 1 correspond to nearest neighbor interpolation and linear interpolation, respectively. However, it was observed that performance deteriorates with interpolation orders above 2, so results are only shown for orders between 0 and 2, i.e., nearest neighbor, linear, and quadratic. Each spline interpolation operates on one SH coefficient at a time.

The interpolation coordinates were set up in accordance with how the low-resolution data were produced. As each low-resolution voxel is obtained by averaging a $2 \times 2 \times 2$ voxel regions in the high-resolution data, its center is placed at the center of said region. For example, and considering data in a single dimension for simplicity, for high-resolution data with coordinates $x_i = i, i \in \{1,N\}$, the corresponding low-resolution data have coordinates $y_j = 2j-0.5, j \in \{1,N/2\}$. These latter coordinates are used to set up the interpolation problem to infer the values at the former coordinates. The interpolation was performed using the ndimage.map_coordinates function of the scipy toolbox^43^ for Python.

### Analyses on synthetic data

In order to assess which interpolation method is able to better represent the underlying structure of the original data, synthetic data was generated from the main fODF peaks of each of the 35 test subjects and a common signal response function. Then, the SH coefficients were obtained via the CSD technique, and the main fODF peaks were singled out using a relative peak threshold of 0.5, a minimum separation angle between peaks of 25° and specifying a maximum of 5 peaks. This peak distribution per voxel acts as the “ground-truth” to be approached by all interpolation methods. In order to simulate characteristics of a real acquisition, noise was added to these “noise-free” (also referred to as “clean”) datasets to obtain signal with $SNR=20$ (defined as $SNR=1/\sigma _g$, where $\sigma _g$ is the standard deviation of the underlying Gaussian noise in the simulated signals). Low-resolution data and upsampled SH coefficients were obtained following the method presented in the previous section for interpolation orders from 0 to 2 and the implemented network. The main peaks were singled out with the same settings previously mentioned and all upsampled distributions were compared to ground truth employing an Earth Mover’s Distance (EMD)^44^ which quantifies the quality of the angular distribution that could be employed in further analyses, such as tractography. The details of this procedure, which was implemented in Python, are described in the Supplementary material. The metric obtained from this implementation of EMD can be interpreted as the total angular “work” required for the upsampled distributions to be equal to the ground truth distribution (the lower the better). Henceforth, the term “angular error” will be used to refer to the angular “work”.

To test the generalization of the implemented model and study the improvement in fODF estimation when upsampling low-resolution data, synthetic data obtained from a different single-shell protocol was generated. The protocol consisted of a single volume with no diffusion weighting and 60 volumes with a b-value of 3000 s/$\hbox{mm}^2$ and diffusion encoding directions arranged according to the tables given by Sloane et al.^45^ Three datasets per test subject were generated: a noiseless high-resolution one, and noisy high-resolution and low-resolution ones. The first two featured the same resolution as the HCP data, while the last one featured twice as large voxels. To simulate the *SNR* gain obtained by acquiring low-resolution data, the high-resolution and low-resolution *SNR*’s (${SNR}_{hr}$ and ${SNR}_{lr}$, respectively) featured the following relation: ${SNR}_{lr}=8\sqrt{2}\,{SNR}_{hr}$. The 8-fold gain represents the larger voxels in low-resolution data, and the other represents the gain obtained by repeating the measurement to boost the *SNR*. ${SNR}_{hr}$ for all test subjects was set to 5, yielding a ${SNR}_{lr} \approx 56.$ The main fODF peaks for both clean and noisy high-resolution data were obtained by directly estimating the SH coefficients and singling out fODF peaks as presented above; the ones for the low-resolution data were obtained estimating the SH coefficients and upsampling them via the implemented network. The main peak distributions obtained for the noisy high-resolution and upsampled low-resolution data were then compared to the distribution obtained for the clean high-resolution data with the EMD approach described above. Moreover, to study the ${SNR}_{hr}$-dependence on the improvement of the fODF’s estimation, the same datasets for a single representative subject featuring ${SNR}_{hr}$ in the 2–20 range were generated and compared as previously stated.

## Results

### SH metrics

The upsampled high-resolution SHs generated by the proposed diffusion super-resolution network (henceforth referred to as DSR) as well as by spline interpolation were compared to the ground truth high-resolution data on a variety of metrics. In addition to measuring overall performance, it was also measured separately for voxels belonging to WM, GM, and CSF, as specified by the parcellation volume available for each subject.

**Fig. 2:**
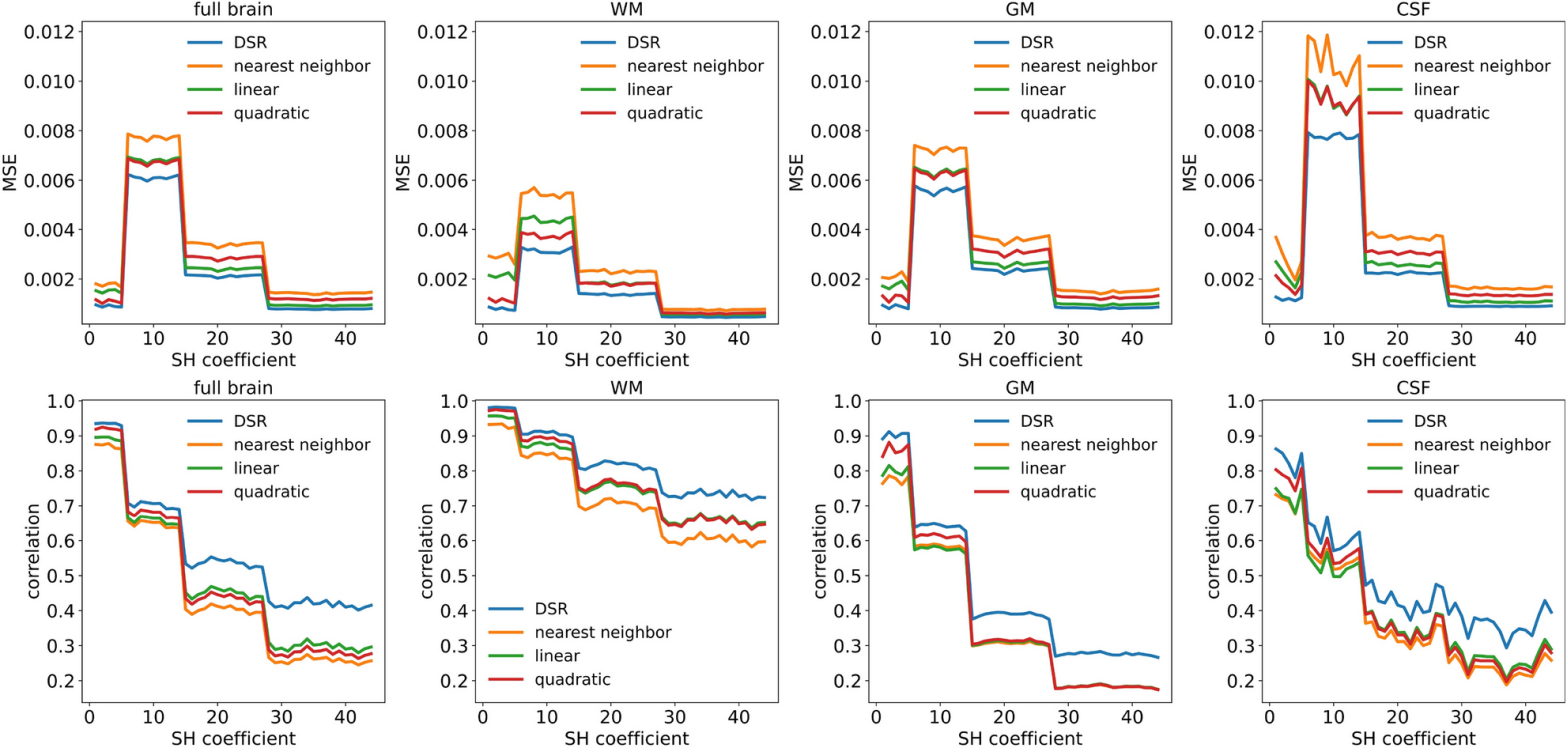
Comparison of similarity metrics between original and upsampled high-resolution SHs, subdivided by metric, tissue type, and SH coefficient. Plotted curves show average metrics for 35 test subjects. Top row: MSE (lower is better). Bottom row: correlation (higher is better). Columns, left to right: overall metrics over the full brain, WM, GM, CSF. MSE: mean squared error, WM: white matter, GM: gray matter, CSF: cerebrospinal fluid.

Figure 2 presents a comparison of mean squared error (MSE, lower is better) and correlation coefficient (higher is better) between the upsampled and ground truth high-resolution data over the SH index, and subdivided by tissue type. The performance for all methods shows a strong dependence on the specific SH coefficient, with four groups of SHs ($Y_{\ell m}$ with $\ell$=2, 4, 6, and 8, respectively) that share a roughly similar performance. For MSE, with the exception of the first group of harmonics, performance seems to improve for higher SH coefficients. However, the performance pattern shown by MSE largely reflects the different value ranges that SHs in the four different groups take, with lower values in the ground truth data resulting in similarly low MSE values. In contrast, the correlation metrics, which are independent of the scale of the data, show that performance for all methods diminishes for higher SH coefficients.

The performance of all methods also shows a clear dependence on tissue type, with superior performance on WM compared to GM and CSF. This is to be expected, as the highly anisotropic axonal tracts in the WM domain are more self-similar across spatial scales than the shorter dendritic fibers in GM. Furthermore, the free water diffusion seen in CSF does not fulfill the assumptions of diffusion along fiber bundles implicit in CSD, which make the generated fODFs highly random in this tissue type.

Both metrics show that DSR outperforms spline interpolation across all SH coefficients and for all tissue types. For the lowest SH coefficients, corresponding to low angular-frequency components of the fODFs, the gain in performance from using DSR compared to spline interpolation of order 2 is comparable to the gain in performance from increasing the spline order by 1 for splines of order 0 and 1. However, the performance gains, in terms of the correlation metric, are much more substantial for the higher SH coefficients, suggesting that DSR far surpasses the other methods at accounting for the higher angular frequencies of the upsampled fODFs. In addition to MSE and correlation, the various methods were also compared on the basis of mean absolute error, structural similarity index, and peak signal-to-noise ratio, all of which showed DSR outperforming spline interpolation methods (additional results can be found in the Supplementary material).

**Fig. 3:**
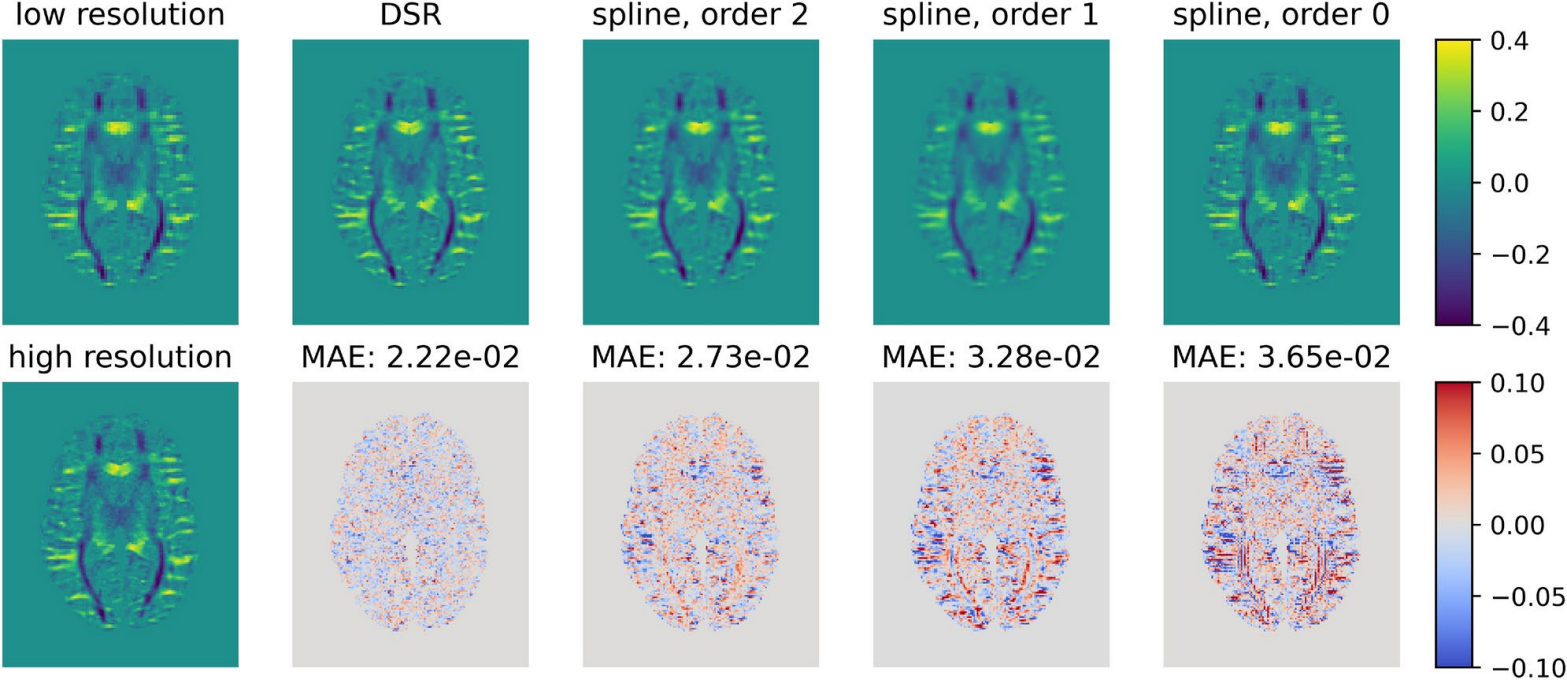
Comparison of upsampling performance of the evaluated methods on the coefficient of the second SH ($Y_{2,0}$) for a single axial slice of a representative test subject. The leftmost plots show the low-resolution image used as the basis for upsampling and the high-resolution ground truth. Top row: upsampled SH coefficients obtained from the various methods. Bottom row: difference maps between upsampled and ground truth SH maps. MAE: mean absolute error (lower is better). DSR upsampling produces the smallest MAE, as well as having the least amount of overt structure in the resulting difference maps. For the other upsampling methods, certain anatomical structures, such as cortical sulci and gyri, and WM fiber bundles, are recognizable in the difference maps, revealing their uneven performance over different brain features.

Figure 3 presents difference maps between the ground truth and upsampled (high-resolution) maps of the second SH coefficient ($Y_{2,0}$) for a single axial slice of one test subject. As can be seen, the maps produced by DSR show the least difference with respect to the ground truth. Moreover, the difference map from DSR shows the least amount of overt spatial structure, having the appearance of uniform noise, compared to those of the other methods, which suggest that DSR is better at accounting for spatial structures present in the data than the other methods, resulting in sharper upsampled maps.

### Reconstructed fODF metrics

The upsampled SH coefficients for all methods were converted back into fODFs and compared to the ground truth fODFs. The effects of the different upsampling methods on the estimated local orientations of fiber bundles were studied by extracting the peaks of the fODFs produced by each method, using a relative peak threshold of 0.5, a minimum separation angle between peaks of 25° and specifying a maximum of 5 peaks.

**Fig. 4:**
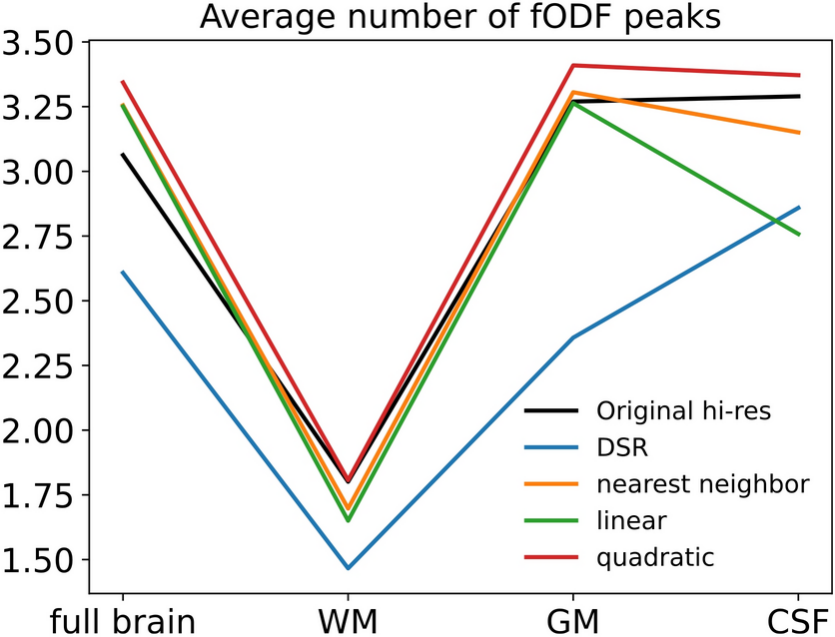
Comparison of average number of fODF peaks present in each tissue type between original high-resolution data and upsampled data. Plotted curve shows average metrics for 35 test subjects.

Figure 4 shows the average number of fODF peaks in the original high-resolution data and upsampled data for each tissue type. While DSR produces substantially fewer peaks than present in the ground truth for all tissue types, other upsampling methods do so only in WM, and overestimate the number of peaks in GM and CSF. It should be noted that due to the nature of the spline interpolation of order 0 (nearest neighbor), these results are also indicative of the metrics obtained with low-resolution data.

**Fig. 5:**
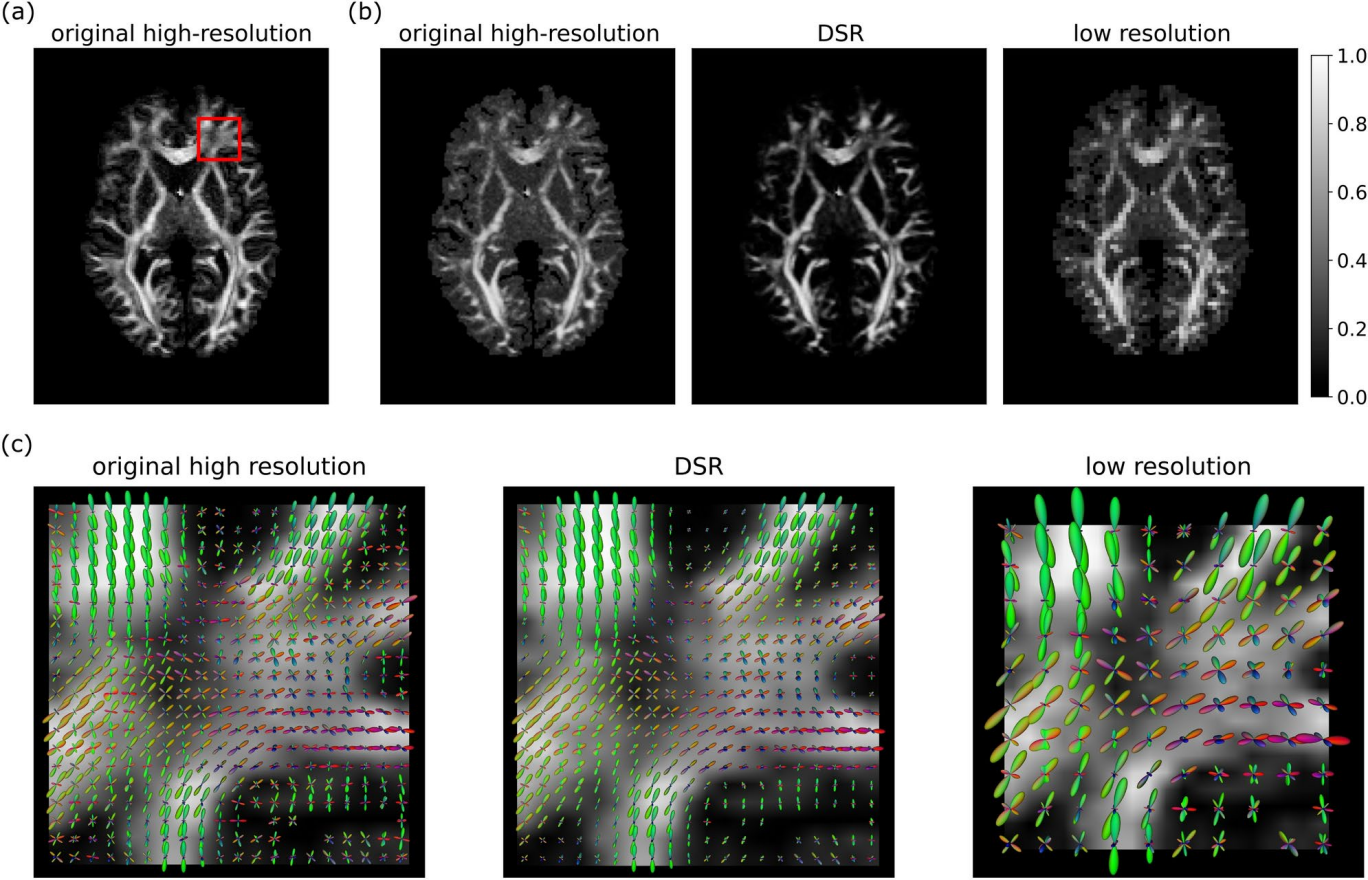
(**a**) Fractional anisotropy (FA) map of the original high-resolution data for a representative test subject. (**b**) Generalized anisotropy (GA)^46^ maps for the original high-resolution data (left), DSR (middle), and low-resolution data (right) for a representative test subject. (**c**) Comparison of fODF glyphs for original high-resolution data and DSR in a region with prominent crossing fibers (red ROI in (**a**)) for a representative test subject. The low-resolution plot in (**c**) presents the same region in the low-resolution data upsampled by DSR. In addition to correctly representing the main fODF orientations, DSR has a denoising effect on small, spurious fODF components, as well as on regions of uncertain orientation such as CSF.

In Fig. 5a and b we illustrate the fractional and generalized anisotropy (FA and GA) maps for a representative subject. Our implementation of the GA index differed from its original formulation^46^ in that the fODF values were taken to be the random variable whose variance was estimated using analytical expressions^14^ in terms of the SH coefficients. This variance ($V_{Y}$) was mapped to the GA index through $\textrm{GA}=\tanh (V_{Y})$. The GA map obtained through the DSR technique exhibited a much clearer delineation of WM from GM areas. This is mostly due to the GA estimated via the DSR method being substantially reduced in GM. The result was a significant improvement in contrast-to-noise ratio making it possible to discern smaller WM innervations.

Figure 5c presents renderings of fODFs for a WM region characterized by crossing fibers. As can be seen, DSR successfully preserves the crossing fibers wherever they are a prominent feature of the data. Furthermore, while there is substantial noise in the ground truth fODFs, with many small peaks present even in areas with a single fiber population, the results of applying DSR are smoother overall, retaining only the most prominent peaks and shrinking the fODFs in CSF regions. Also shown is a rendering of the same region in the low-resolution data used as upsampling input, highlighting the increase in gains in spatial specificity of fiber orientation from using DSR.

### Synthetic data

**Fig. 6:**
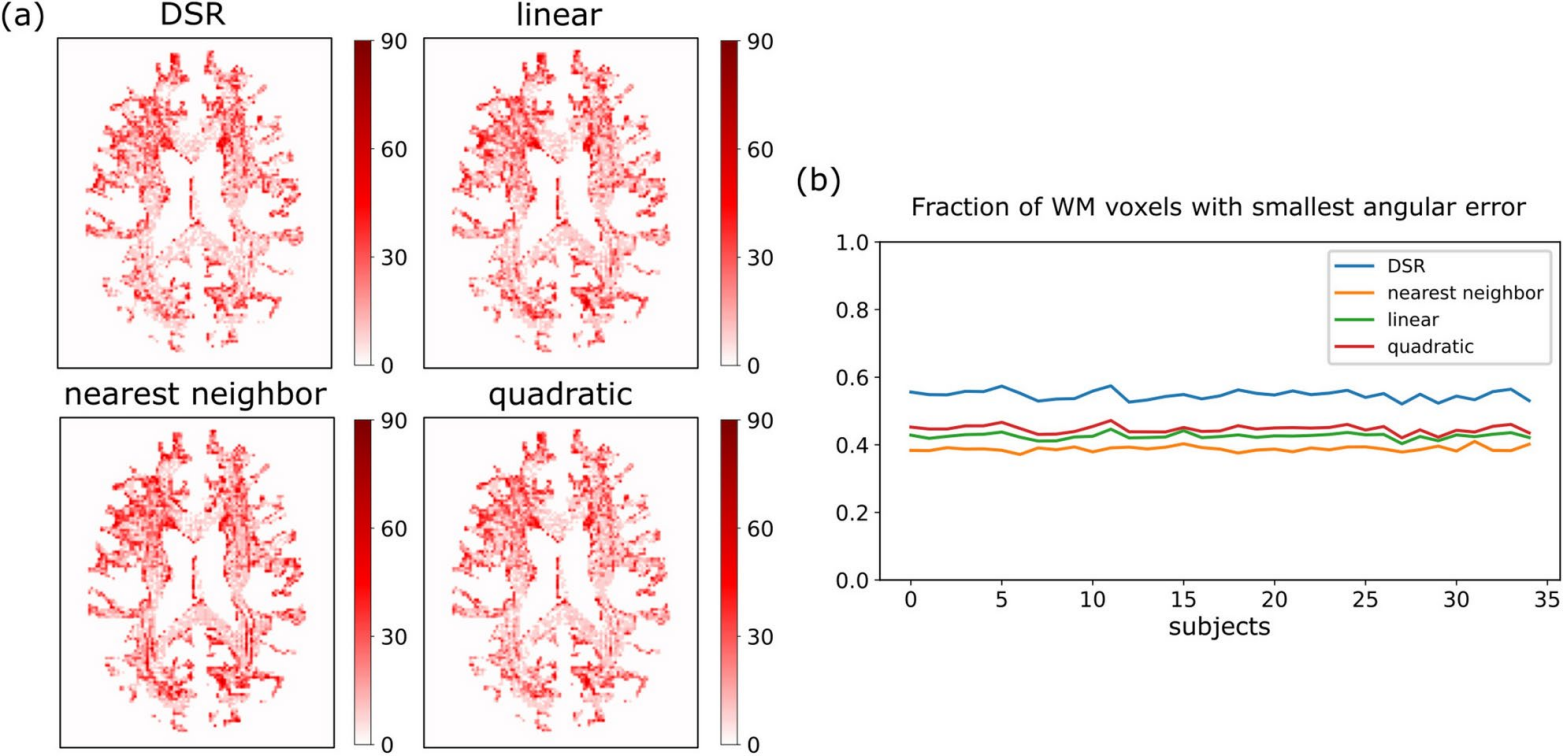
Comparison of angular error obtained for different interpolation methods. (**a**) Angular error maps in white matter tissue of a representative subject for different interpolation methods. (**b**) Fraction of total WM voxels for which the smallest error was obtained by each interpolation method, for each of the test subjects. Note that the total fraction sum for all interpolation methods is not equal to 1 since multiple methods achieved the smallest error in the same voxel. See the Supplementary material for further details regarding this fraction.

Figure 6 shows the EMD measure indicating the dissimilarity between the fiber orientations of the ground truth synthetic data and those obtained via various upsampling methods. The angular error maps over white matter voxels show slight differences between all interpolation methods, however it should be noted that DSR manages to correctly interpolate a larger number of voxels’ fODF peak distribution. This is more clearly shown after tallying up the number of white matter voxels where each interpolation method obtained the smallest angular error for each test subject; it can be observed that DSR consistently performs the best when compared to the other interpolation methods. Note that for a given voxel, multiple interpolation methods achieve the same smallest error (i.e., 0) and were all marked as the “best” method for that voxel. Thus, the fractions presented in Fig. 6b do not sum up to 1 for each test subject (see the Supplementary material for further details on the calculation of the presented fraction).

**Fig. 7:**
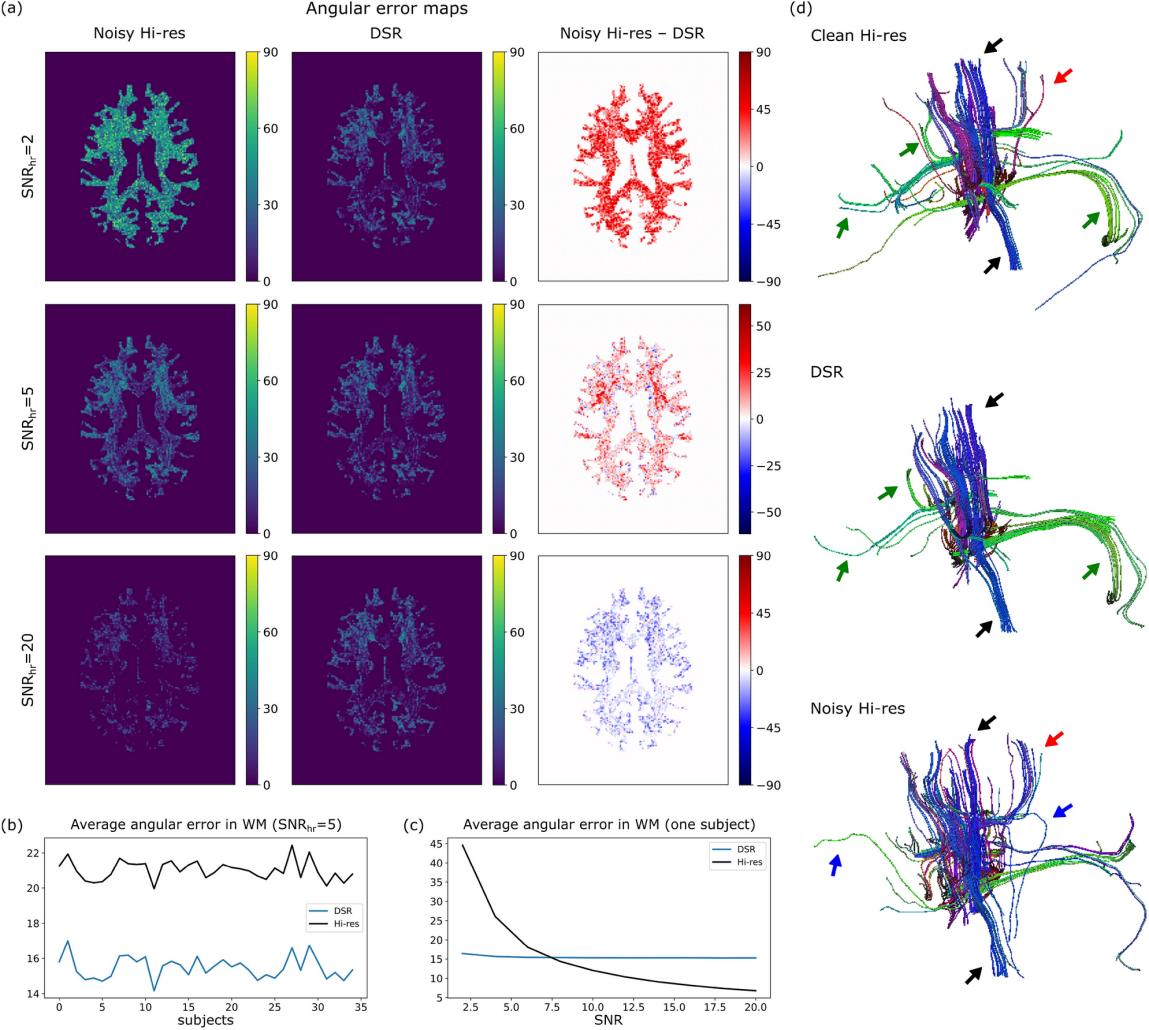
Improvement on fODF estimation. (**a**) Angular error maps for noisy high-resolution data (left), upsampled high-resolution data obtained via DSR (middle), and the difference between the two (right), for ${SNR}_{hr}=2$ (top), 5 (middle), and 20 (bottom). (**b**) Average error in white matter (WM) voxels for synthetic data with ${SNR}_{hr}=5$ for each subject. (**c**) Average error in WM voxels for a representative subject and different ${SNR}_{hr}$’s. (**d**) Tracts obtained by seeding the voxels with the highest angular error difference for synthetic data with ${SNR}_{hr}=5$ in “noise-free” (clean) data (top), upsampled high-resolution data obtained via DSR (middle), and noisy high-resolution data (bottom). Black arrows point towards main tracts obtained from all three datasets; green arrows to tracts observed in the clean and upsampled datasets but not in the noisy high-resolution one; red arrow to a tract observed in the clean and noisy high-resolution datasets but not in the DSR upsampled one; and blue arrows to tracts observed in noisy high-resolution dataset but not in the clean high-resolution or DSR upsampled ones.

The improvements in the estimates of fODFs’ main peaks are presented for various levels of noise in Fig. 7. The angular error maps for ${SNR}_{hr}=2$ show a vast improvement in fODF estimation featuring high angular errors across white matter voxels. For ${SNR}_{hr}=5$, the improvement is less pronounced yet still evident over all white matter voxels. The improvement for this ${SNR}_{hr}$ is consistent across all test subjects, featuring an average reduction of angular error of approximately 6° as shown in Fig. 7b. On the other hand, for ${SNR}_{hr}=20$, the estimates obtained via DSR are more accurate over small areas of white matter voxels. This is more apparent after computing the average angular error in white matter tissue of a representative subject for different ${SNR}_{hr}$’s. As shown in Fig. 7c, employing upsampled low-resolution data with DSR features an improvement in fODF peak estimation (lower angular error) over employing high-resolution data up until ${SNR}_{hr}\approx 7.5$.

### Tractography on synthetic data

One of the principal uses of fODFs obtained through CSD involves precise estimation of fiber orientations for tractography, which typically relies on a continuous approximation to the field of fiber orientations. Thus, an accurate estimation of fiber orientations is expected to improve the performance of tractography algorithms. To examine this possibility, streamlines were generated from the clean synthetic data with ${SNR}_{hr}=5$ for high-resolution noisy, and upsampled low-resolution noisy datasets. The streamlines were generated with the EuDX algorithm^47^ in dipy with default parameters and seeds placed on white matter voxels featuring the largest differences in the noisy high-resolution and upsampled low-resolution angular error maps. Tracking was stopped when the streamlines exit the white matter. The resulting tracts are shown in Fig. 7d, where it can be observed that the upsampled data obtained via DSR features most tracts resembling the ones in the clean high-resolution data (black arrows). The tracts obtained from noisy high-resolution data also resemble the ones from clean high-resolution data (black arrows), however with very noticeable variations in the direction of the tracts and even spurious tracts (blue arrows). It should also be noted that a few tracts present in clean high-resolution data are obtained only via upsampling with DSR (green arrows), and at least one bundle is obtained only via the noisy high-resolution data (red arrow). Additionally, we calculated fidelity metrics to asses the quality of the tractography results obtained from these two datasets (low-resolution upsampled via DSR and noisy high-resolution). The following metrics were computed by employing the scilpy toolbox which is part of the Tractometer framework^48^: the voxel-wise angular correlation, the density correlation and streamline distance in a neighborhood of voxels, tracts’ overlap, overreach, and Dice score. Here, the tracts obtained with the clean dataset were treated as ground truth in all comparisons. The tractography results obtained from low-resolution data upsampled via DSR obtained better scores compared to other upsampling approaches for some metrics, while quadratic interpolation provided similar results for some metrics (see the Supplementary material for further details on the metrics and results).

## Discussion

Throughout the presented experiments, DSR has shown improved upsampling performance over spline interpolation methods (see Figs.  2, 3, 4 and 6). However, the learning-based nature of DSR’s upsampling, coupled with the large training corpus used, gives it substantial advantages over ordinary interpolation methods that go beyond the raw metrics. First, it allows DSR to conform its upsampling approach to local anatomical brain features, resulting in sharper upsampled maps (see Fig.  3). Furthermore, it allows DSR to distinguish those aspects of the data which are coherent across its training dataset, such as the orientations of the main fODF peaks, from those that are not coherent, such as spurious fODF peaks caused by the high noise-susceptibility of CSD. This latter feature causes DSR to have a denoising effect on the upsampled fODFs, allowing it to remove spurious fODF components that are present in the high-resolution ground truth while preserving the relevant orientation information (see Fig.  5). Remarkably, it is capable of doing this while using only 1/8 of the data available in the ground truth. A possible explanation for this is that our DSR approach uses the information in all 44 SH coefficients concurrently, while the spline interpolation operates on one SH coefficient at a time. Furthermore, the results in Fig. 7 suggest the feasibility to generate higher fidelity estimates of the distributions of fODF peaks when employing upsampled data via DSR instead of noisy high-resolution data with ${SNR}_{hr}<7.5,$ and consequently more accurate tractography results. This is observed on the tracts obtained from upsampled data, which do not show any false positives, (i.e., tracts not present in the clean dataset), whereas this is not the case for the tracts obtained from noisy high-resolution data. This is further observed in the calculated overlap and overreach fidelity metrics, for which the upsampled data via DSR outscored the noisy high-resolution data. However, as shown by the comparison between high-resolution synthetic and upsampled data via all methods, not all main fODF peaks are correctly recovered or interpolated. This suggests that upsampling methods, however sophisticated they may be, should not be expected to replace high-resolution acquisitions without some information being lost in the process. However, our simulations suggest that in low SNR conditions the high-resolution scans perform poorly compared to DSR employed on low-resolution data.

Repeated application of the DSR method to obtain images with voxel size down to $156\,\upmu\hbox{m}$ yields sharp scalar maps with a remarkable level of spatial detail (see Fig. 4 in the Supplementary material). Interestingly, repeated application of the DSR method provides a progressive reduction in the number of fiber directions detected within each voxel. Importantly, we do not claim that these are the features that one would expect from high-resolution images. In fact, Schilling et al.^49^ have observed just the opposite, i.e., an increase in the number of fiber orientations at higher resolutions. Rather, the reduction we have observed is possibly due to: (i) the network learning the persistent features that are more robust to noise, which leads to a reduction of artifactual peaks, and (ii) the method’s ability to assign peaks of the original fODF to different parts of the “low-res” voxel. Here, by “peaks” we refer to those that are possible to resolve, given the limitations involving the angular resolution of dMRI at the acquisition resolution as well as the motional averaging of the diffusion process.

This study contains certain limitations such as the generalization aspect of the trained network. To use the network for diseased subjects, for data collected using another MR scanner, another sequence or another resolution, would probably require the acquisition of additional data and re-training the network, which is not explored in this study. However, the robustness of the model shown by the results in synthetic data is encouraging on this front, not only because new (although with high resemblance to the original) validation volumes were employed, but also because SH coefficients were generated with a different acquisition protocol from the HCP dataset. Secondly, the network uses limited spatial information when training which may be considerably impactful in complicated areas of the brain (with hard to track fibers). For example, it may be conceivable to design a network which is only trained on data from a certain tissue type or region to improve its performance in these areas of the brain.

We note that our approach can be extended and generalized in a number of ways. The training performed on the patches of fODFs could be performed on diffusion-weighted data^30^, which could feature other sampling schemes such as those that cover a larger region of the sampling space^13^; performing it on the spherical harmonics series helped us avoid issues related to data harmonization. A basis other than the SH basis could be employed for representing such data^50^. The input and/or the output could include scalar measures of tissue structure^51^. A representation of asymmetric ODFs^52–54^ could be employed that could allow disambiguation of asymmetric features that are encountered for bending fibers or Y-shaped crossings. Regarding using a larger spatial region of interest ($5 \times 5 \times 5$), this can possibly be achieved by adding Dropout regularization between the dense layers to prevent overfitting.

## Conclusion

We have presented a deep-learning-based super-resolution approach for upsampling diffusion fODF data. The proposed method outperforms standard spline interpolation methods, and is capable of rendering nuanced anatomical features in scalar maps derived from the fODF data. We believe that the proposed method can be particularly useful for improving tractography, as it inherently relies in interpolation for estimating the local orientation of fiber tracts in positions between the available voxels’ centers. We have shown that rather than collecting high-resolution data with signal-to-noise ratios below 7.5, one could collect low-resolution data at a fraction of the acquisition time and obtain higher-fidelity fODF distributions, consequently tractography results, via the super-resolution estimation framework.

## Supplementary Information

Supplementary Information.

## Data Availability

The MRI datasets used in the current study are available in the WU-Minn Human Connectome Project^38^ repository at db.humanconnectome.org/data/projects/HCP_1200. Specifically, we used preprocessed structural and diffusion MRI data from the “100 Unrelated subjects” sub-group. Subjects 101309, 108828, 131217, 131722, and 189450 were excluded from this work due to slightly incomplete coverage of the white matter by the diffusion MRI data. To facilitate reproducibility and extension of this work, the used code can be shared upon request.
