# Supplementary material for: Super-resolution mapping of anisotropic tissue structure with diffusion MRI and deep learning
Source: Sci Rep. 2025 Feb 24;15:6580. doi: 10.1038/s41598-025-90972-7 (PMC11850900; doi:10.1038/s41598-025-90972-7)
Supplement: Supplementary file 1 — Supplementary Information. [file 41598_2025_90972_MOESM1_ESM.pdf]

Super-resolution mapping of anisotropic tissue structure with diffusion MRI and deep learning – Supplementary Material

Alfredo Ordinola[1,†], David Abramian[1,2,†], Magnus Herberthson[3], Anders Eklund[1,2,4,+], and Evren Özarslan[1,2,*,+]

[1]Department of Biomedical Engineering, Linköping University, Linköping, Sweden
[2]Center for Medical Image Science and Visualization, Linköping University, Linköping, Sweden
[3]Department of Mathematics, Linköping University, Linköping, Sweden
[4]Department of Computer and Information Science, Linköping University, Linköping, Sweden
[*]evren.ozarslan@liu.se
[†]These authors contributed equally to this work: Alfredo Ordinola and David Abramian.
[+]These authors jointly supervised this work: Anders Eklund and Evren Özarslan.

ABSTRACT

Diffusion magnetic resonance imaging (diffusion MRI) is widely employed to probe the diffusive motion of water molecules within the tissue. Numerous diseases and processes affecting the central nervous system can be detected and monitored via diffusion MRI thanks to its sensitivity to microstructural alterations in tissue. The latter has prompted interest in quantitative mapping of the microstructural parameters, such as the fiber orientation distribution function (fODF), which is instrumental for noninvasively mapping the underlying axonal fiber tracts in white matter through a procedure known as tractography. However, such applications demand repeated acquisitions of MRI volumes with varied experimental parameters demanding long acquisition times and/or limited spatial resolution. In this work, we present a deep-learning-based approach for increasing the spatial resolution of diffusion MRI data in the form of fODFs obtained through constrained spherical deconvolution. The proposed approach is evaluated on high quality data from the Human Connectome Project, and is shown to generate upsampled results with a greater correspondence to ground truth high-resolution data than can be achieved with ordinary spline interpolation methods. Furthermore, we employ a measure based on the earth mover's distance to assess the accuracy of the upsampled fODFs. At low signal-to-noise ratios, our super-resolution method provides more accurate estimates of the fODF compared to data collected with 8 times smaller voxel volume.

Additional results

Similarity metrics

The mean squared error (MSE), standard deviation of the squared error (STDSE), mean absolute error (MAE), standard deviation of the absolute error (STDAE) and correlation similarity metrics averaged over all subjects and subdivided by tissue type and spherical harmonic (SH) coefficients are presented in Supplementary Figure 1, and the same metrics averaged over all SH coefficients and subdivided by tissue type and test subjects are presented in Supplementary Figure 2. The self similarity index measure (SSIM) and peak signal-to-noise ratio (PSNR) similarity metrics are presented in Supplementary Figure 3. From all these results it can be observed that DSR interpolation performs consistently the best across all test subjects and brain regions.

Repeated upsampling

In order to explore the limitations of DSR upsampling, as well as to evaluate its performance at upsampling resolutions it was not specifically trained on, we experimented with upsampling the original high-resolution data with DSR. To this end, the data was upsampled repeatedly, by feeding the output of DSR back as input. This upsampling was repeated three times, being very computationally-demanding. As there is no ground truth for the super-resolved data, we can only perform a qualitative evaluation of the results. Supplementary Figure 4 presents plots of the magnitudes of the first three fODF peaks, as well as of the overall number of peaks per voxel, for the super-resolved data. Repeated upsampling does not result in blurring of the data, as could be expected, but actually produces sharp spatial maps with a high level of detail, albeit showing some streaking and checkerboard artifacts on the higher resolutions. Some notable features can be seen in Supplementary Figure 4d, where long, single-voxel-wide regions of crossing fibers are well-resolved under repeated upsampling, with some features retaining a width

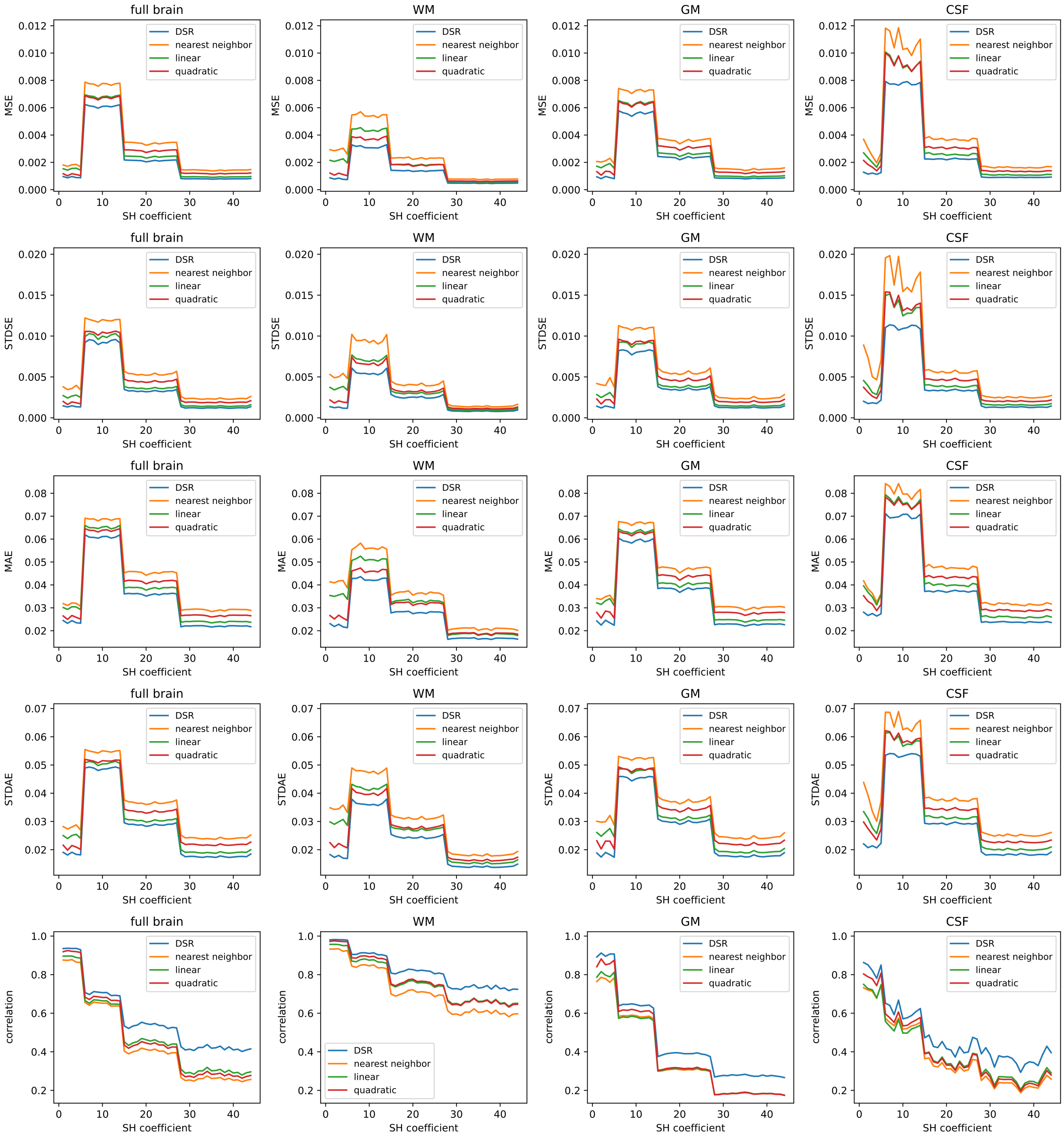

Supplementary Figure 1. Comparison of similarity metrics between original and upsampled high-resolution SHs, subdivided by metric, and SH coefficient. Plotted curves show average metrics for the 35 test subjects. Rows, top to bottom: MSE (lower is better), STDSE (lower is better), MAE (lower is better), STDAE (lower is better), and correlation (higher is better). Columns, left to right: overall metrics over the full brain, WM, GM, CSF. MSE: mean squared error, STDSE: standard deviation of the squared error, MAE: mean absolute error, STDAE: standard deviation of the absolute error, WM: white matter, GM: gray matter, CSF: cerebrospinal fluid.

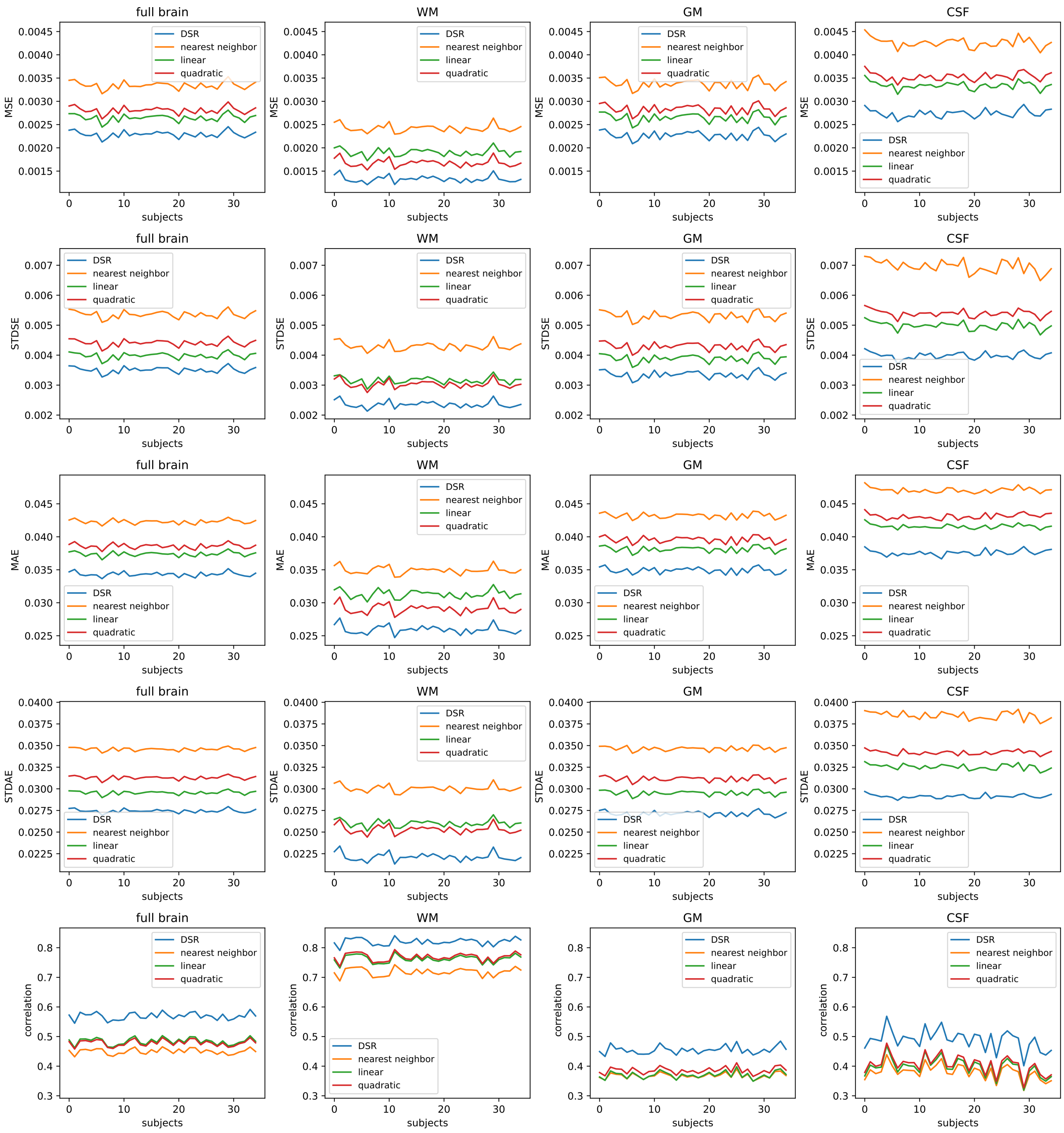

Supplementary Figure 2. Comparison of similarity metrics between original and upsampled high-resolution SHs, subdivided by metric, and subject. Plotted curves show average metrics for all SHs. Rows, top to bottom: MSE (lower is better), STDSE (lower is better), MAE (lower is better), STDAE (lower is better), and correlation (higher is better). Columns, left to right: overall metrics over the full brain, WM, GM, CSF. MSE: mean squared error, STDSE: standard deviation of the squared error, MAE: mean absolute error, STDAE: standard deviation of the absolute error, WM: white matter, GM: gray matter, CSF: cerebrospinal fluid.

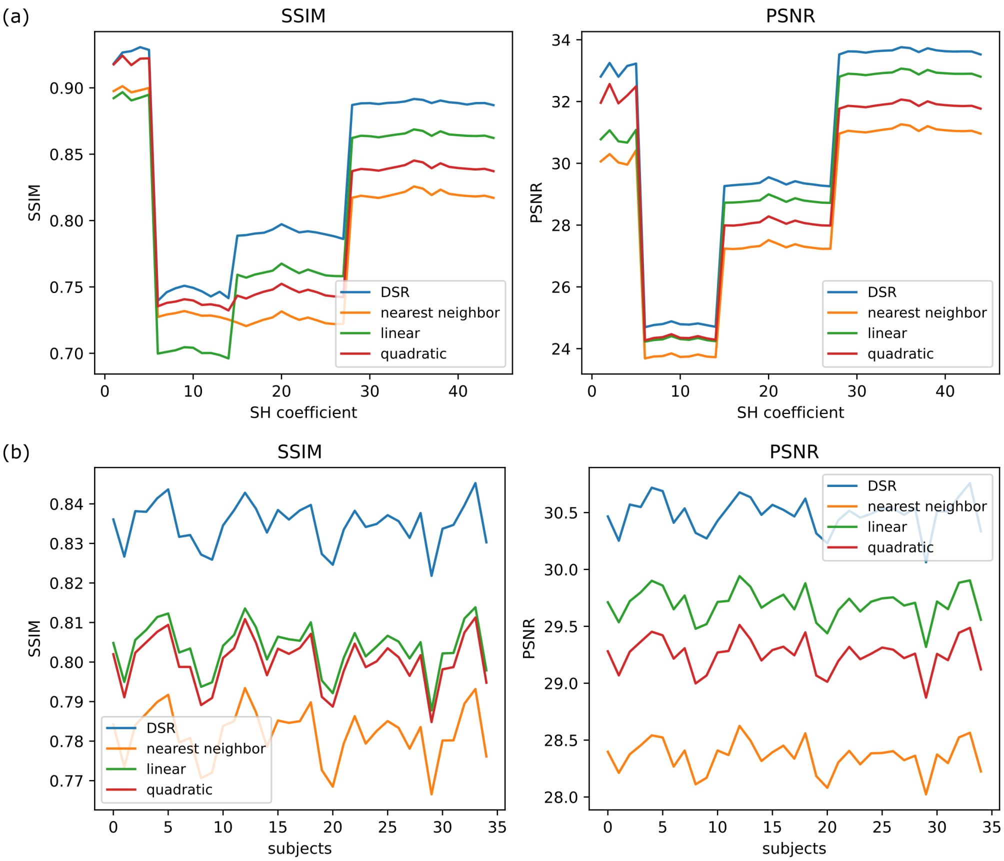

Supplementary Figure 3. Comparison of similarity metrics between original and upsampled high-resolution SHs, subdivided by metric, and (a) SH coefficients (averaged over all test subjects) and (b) test subjects (averaged over SHs). Left column: SSIM (higher is better). Right column: PSNR (higher is better). SSIM: similarity index measure, PSNR: peak signal-to-noise ratio.

of a single voxel in the highest achieved resolution.

The denoising effects of DSR are also made apparent under repeated upsampling. It can be seen that the magnitude of the first and second fODF peaks is generally preserved or somewhat amplified in WM, where it is well-specified. On the other hand, the magnitudes of all fODF peaks are attenuated with each upsampling in regions outside WM, where the assumptions of CSD do not fully apply, and the self-similarity of diffusion orientation across scales is lower. This is especially apparent for the third fODF peak, which is rarely detected in WM, and is almost completely suppressed after upsampling three times. The overall number of fODF peaks per voxel is also reduced, as shown in Supplementary Figure 4. Please see the main manuscript for a discussion on the reduction of the number of peaks.

Additional results from synthetic data

Method comparison in white matter

In order to assess the performance of all interpolation methods on different areas of white matter (WM) in the brain, the mean angular error, obtained via the Earth Mover's Distance (EMD) approach presented in the main article, was computed for: (i) voxels corresponding to all of WM, (ii) voxels corresponding only to tracts going through the corpus callosum (CC), and (iii) voxels corresponding to WM wherein the fibers do not go through the CC. The regions corresponding to WM and the CC were obtained via parcellation volumes provided along with the HCP dataset. Recall that the angular error was computed by comparing each upsampled fODFs with the ground truth fODFs, both of which were obtained from synthetic data, and is essentially a similarity metric between distributions of main fODF peaks. The mean angular errors for each test subjects in the three mentioned brain regions are presented in Supplementary Figure 5. It can be observed that DSR performs best in all three analysed regions, however not by a large margin, especially in WM voxels, where spline interpolation of order 2 (quadratic) perform almost as equally good as DSR. Furthermore, the angular error is much smaller in voxels corresponding to tracts going through the CC for all interpolation methods, suggesting the fODFs here are easier to interpolate even when employing nearest neighbor interpolation. This is likely due to the large size of these tracts which would result in a coherent structure over a large area. Other areas of WM include tracts which are significantly smaller, and thus much harder to recover via interpolation, however we reiterate that there is an overall improvement on the angular error (and thus fODF estimation) when employing DSR even in areas which do not include tracts going through the CC.

Tractography fidelity metrics

In order to quantitatively assess the quality of the tractography results obtained from noisy high-resolution data, and upsampled noisy low-resolution data employing different interpolation methods (diffusion super-resolution, nearest neighbor, linear and quadratic), multiple fidelity metrics were calculated. These metrics were: angular correlation, density correlation, distance between streamlines, overlap, overreach, and Dice score. These were calculated by treating the tracts obtained from the clean high-resolution data as ground truth.

The angular correlation measures the voxel-wise similarity between the function evaluated on a sphere derived from the streamlines within the voxel of the ground truth and the one obtained from the assessed method. The density correlation measures the correlation between a neighborhood of voxels in the fiber density maps (i.e., number of streamlines that go through each voxel) of two sets of streamlines; the distance between streamlines is the average distance between two sets of streamlines in a neighborhood of voxels. We note that this last metric is normalized so that it features a range of [0, 1] by first subtracting the minimum distance from all voxels and then dividing this quantity by the difference between the maximum and minimum distance in the whole group of computed tracts. For the overlap, overreach, and Dice score, a binary mask was created that distinguishes voxels that contain at least one streamline (i.e., the object) from voxels without any streamlines (i.e., the background), leading to a binary classification analysis. The overlap measures the proportion of true positives with respect to the total object voxels in the ground truth mask; the overreach measures the proportion of false positives with respect to the total number of object voxels in the ground truth mask; and the Dice score measures the similarity between the ground truth mask and the one obtained from the method to asses (noisy high-resolution and upsampled with different interpolation methods).

Two different sets of streamlines were obtained employing deterministic tractography: for the first, only voxels corresponding to the corpus callosum (CC) were seeded, while for the second, all white matter (WM) voxels were seeded. For both, tracking was stopped when the streamlines exit the WM. The fidelity metric maps obtained from noisy high-resolution data and data upsampled via DSR for the first set of streamlines are presented in Supplementary Figure 6, and for the second set of streamlines in Supplementary Figure 7. The metrics obtained from data upsampled via each interpolation method are shown in Supplementary Table 1, where the mean and standard deviation of the angular correlation, density correlation and streamline distance were calculated over all valid voxels in each set of streamlines (i.e., voxels which feature at least one streamline in the two compared tractographs: the ground truth one, and the one obtained from interpolated or noisy high-resolution data) for all

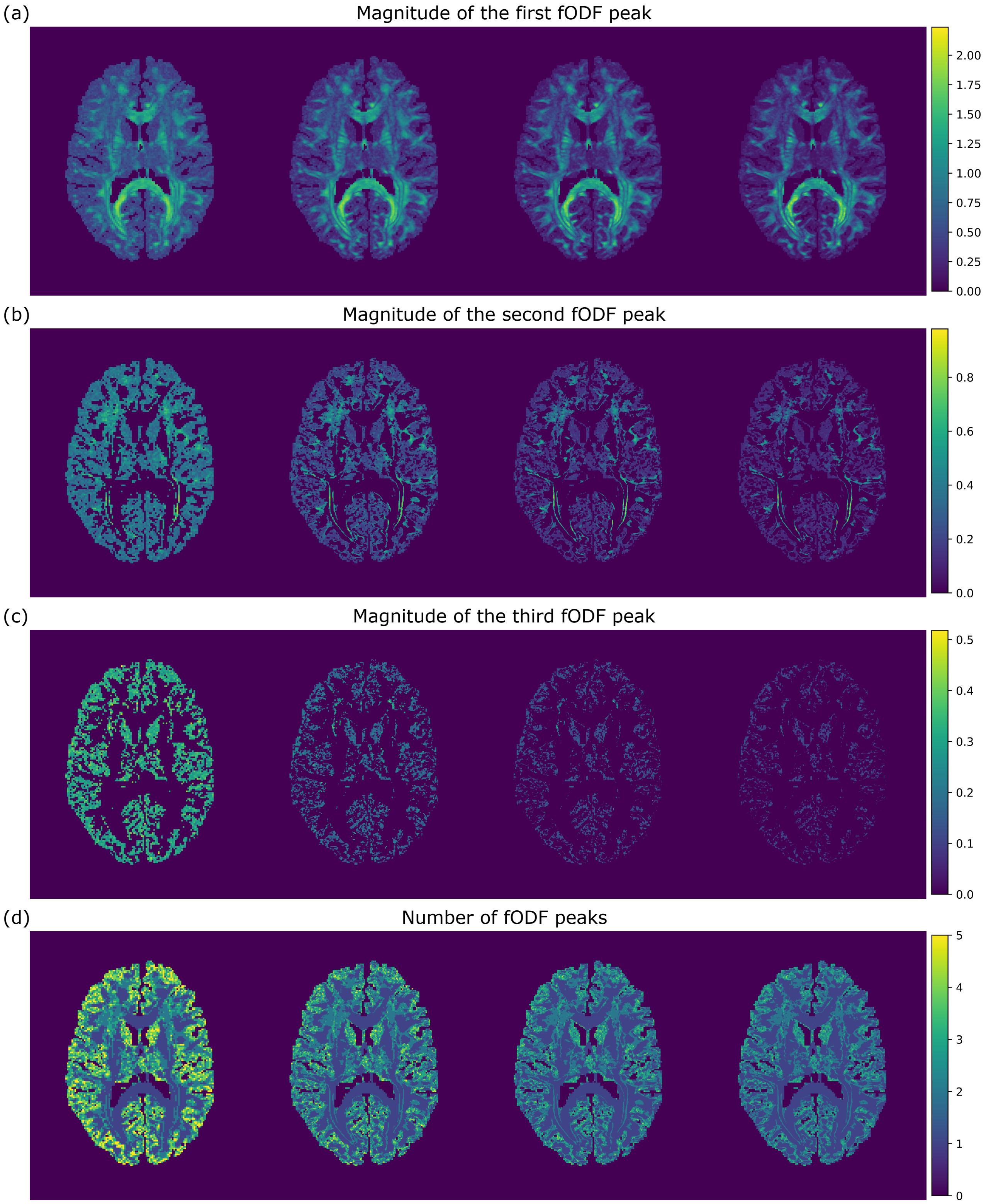

Supplementary Figure 4. Results of repeated upsampling with DSR of the original high-resolution data. The leftmost volume in each row is produced from the original high-resolution data, with each subsequent volume having half the size in each axis of the previous. (a) Magnitude of the first fODF peak. (b) Magnitude of the second fODF peak, where present. (c) Magnitude of the third fODF peak, where present. (d) number of fODF peaks per voxel. In all cases, DSR is capable of generating sharp results with a remarkable level of anatomical detail. Voxel sizes, from left to right: 1.25 mm, 0.625 mm, 0.313 mm, 0.156 mm.

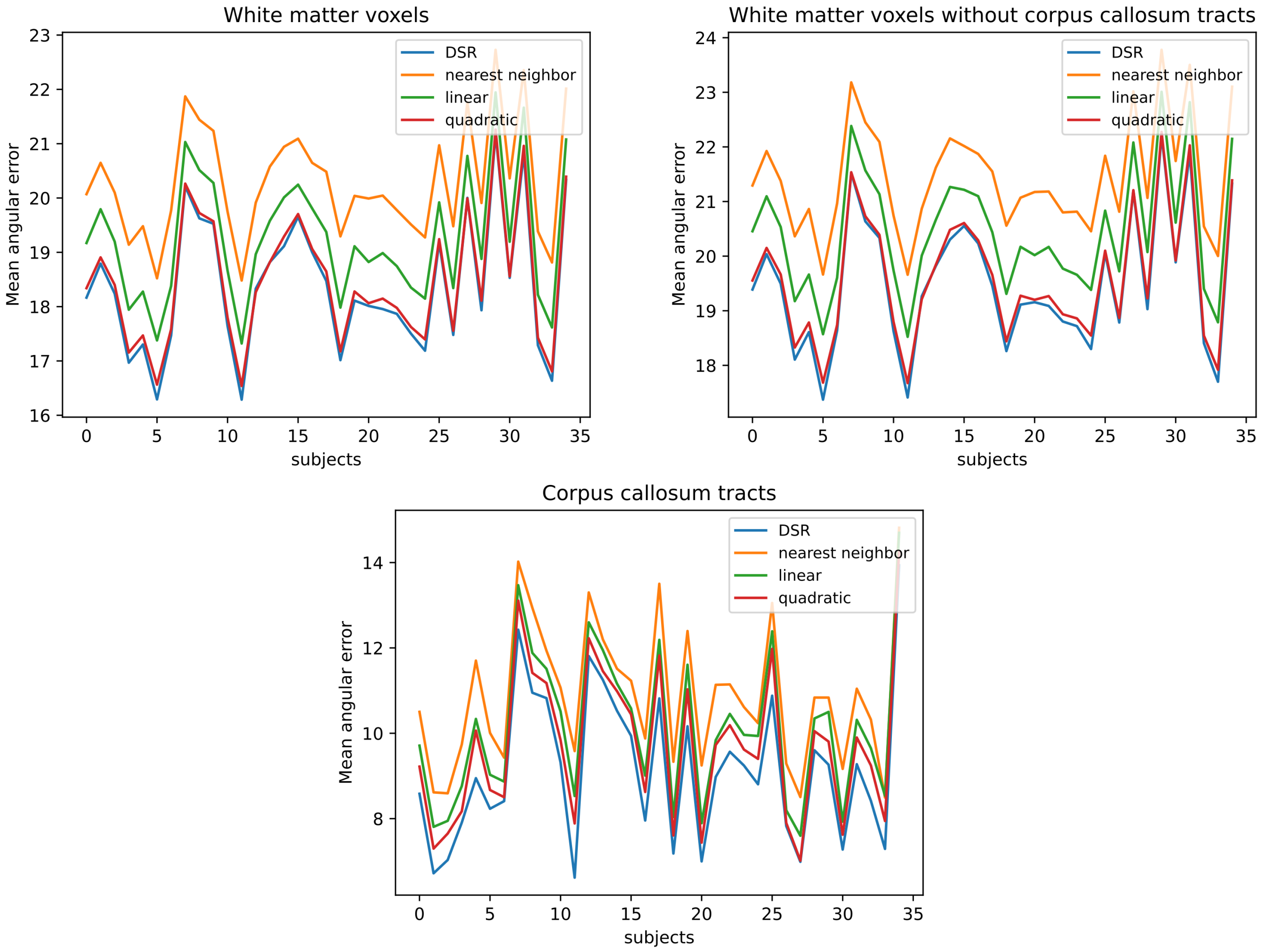

Supplementary Figure 5. Comparison of mean angular error of the main fODF peak distribution (the lower the better) for all interpolation methods on all WM voxels (top left), voxels that correspond to CC tracts (bottom), and WM voxels except the ones corresponding to CC tracts (top right). WM: white matter, CC: corpus callosum.

subjects. We note that the distribution of these metrics over the whole volume is not Gaussian, and thus the interquartile range of some of the reported results are greater than 1.

From the obtained results it can be clearly observed that the tracts obtained from the upsampled data via DSR outscore the tracts obtained from noisy high-resolution data and data upsampled with every other interpolation method, except for the overreach obtained in the first set of tracts with nearest neighbor interpolation, which outscores DSR by a small margin. Nevertheless, these results suggest that tracts obtained via DSR resemble the tractography results one would obtain from clean high-resolution data the most out of all other studied cases.

We should note that all datasets feature a similar performance if one considers only the overlap, overreach, and Dice score metrics for the tracts obtained by seeding the whole WM. However, this was expected as each seed would yield at least one streamline, guaranteeing an almost full streamline coverage of WM areas, and consequently almost perfect scores in the aforementioned metrics. For this case in particular, the other three reported metrics (angular correlation, density correlation and distance between streamlines) are more suitable to compare different methods. Therefore, the overlap, overreach, and Dice score for whole WM seeding are not shown in Supplementary Table 1 to avoid any misinterpretations of these values. This is not the case for the tracts obtained by seeding only the CC. As the tracts can exit the WM, it is possible that the tracts take different paths depending on the upsampled SHs obtained with different interpolation methods. Therefore, the overlap, overreach, and Dice score metrics quantify (albeit indirectly) how close these paths are to the ones in the ground truth (i.e., synthetic high-resolution) data.

Supplementary Table 1. Fidelity metrics obtained for the two sets of streamlines (seeding voxels only from the corpus callosum, and from all white matter voxels) obtained from noisy high-resolution data (None) and different interpolation methods (DSR: diffusion super-resolution network, NN: nearest neighbor, LIN: linear, INT2: quadratic). The mean and standard deviation of the angular correlation, density correlation and streamline distance were calculated over all valid voxels. The best scores for each metric (in terms of their mean value) are in bold. Values shown as $*$ are not significant to the analysis.

Seeds	Int. Method	Ang. Corr.	Dens. Corr.	1− Dist.	Dice	Overlap	Overreach
CC	None	0.79 ± 0.24	0.41 ± 0.29	0.69 ± 0.16	0.68 ± 0.02	0.66 ± 0.02	0.27 ± 0.02
	DSR	$\mathbf{0.88 \pm 0.18}$	$\mathbf{0.49 \pm 0.28}$	$\mathbf{0.76 \pm 0.15}$	$\mathbf{0.75 \pm 0.01}$	$\mathbf{0.72 \pm 0.02}$	0.22 ± 0.01
	NN	0.85 ± 0.20	0.41 ± 0.30	0.72 ± 0.16	0.70 ± 0.02	0.66 ± 0.02	$\mathbf{0.21 \pm 0.02}$
	LIN	0.87 ± 0.19	0.44 ± 0.30	0.74 ± 0.15	0.73 ± 0.02	0.71 ± 0.02	0.23 ± 0.02
	INT2	$\mathbf{0.88 \pm 0.18}$	0.48 ± 0.27	0.75 ± 0.12	0.74 ± 0.02	$\mathbf{0.72 \pm 0.02}$	0.22 ± 0.02
WM	None	0.71 ± 0.31	0.69 ± 0.26	0.82 ± 0.12	$*$	$*$	$*$
	DSR	$\mathbf{0.85 \pm 0.22}$	$\mathbf{0.81 \pm 0.19}$	$\mathbf{0.86 \pm 0.12}$	$*$	$*$	$*$
	NN	0.79 ± 0.26	0.74 ± 0.24	0.81 ± 0.12	$*$	$*$	$*$
	LIN	0.81 ± 0.25	0.78 ± 0.21	0.81 ± 0.11	$*$	$*$	$*$
	INT2	0.83 ± 0.23	0.79 ± 0.19	0.82 ± 0.11	$*$	$*$	$*$

Fraction of white matter voxels with lowest angular error

In order to observe the performance of different interpolation methods across different subjects, the number of white matter voxels with the lowest angular error were tallied up and compared. For a given subject and interpolation method “1”, the fraction employed for this comparison is defined as:

$$f_1 = \frac{\text{Number of WM voxels featuring the lowest angular error with interpolation method “1”}}{\text{Total number of WM voxels}} \tag{1}$$

If a single interpolation method were to feature the lowest angular error for each voxel, it would follow that for a collection of interpolation methods the sum of fractions would be equal to 1. However, in the results obtained in our analysis, multiple interpolation methods yielded the lowest angular error possible (i.e., 0) for the same voxel in multiple occasions. Consequently, the resulting fractions shown in panel (b) of Figure 6 of the main article, do not sum up to 1.

Dissimilarity of two sets of fiber orientations based on the Earth Mover’s Distance

The Earth Mover’s Distance (EMD) is a dissimilarity metric often employed to compare two different distributions. This method was employed in the main article to compare two sets of main fiber orientation distribution functions (fODFs), which

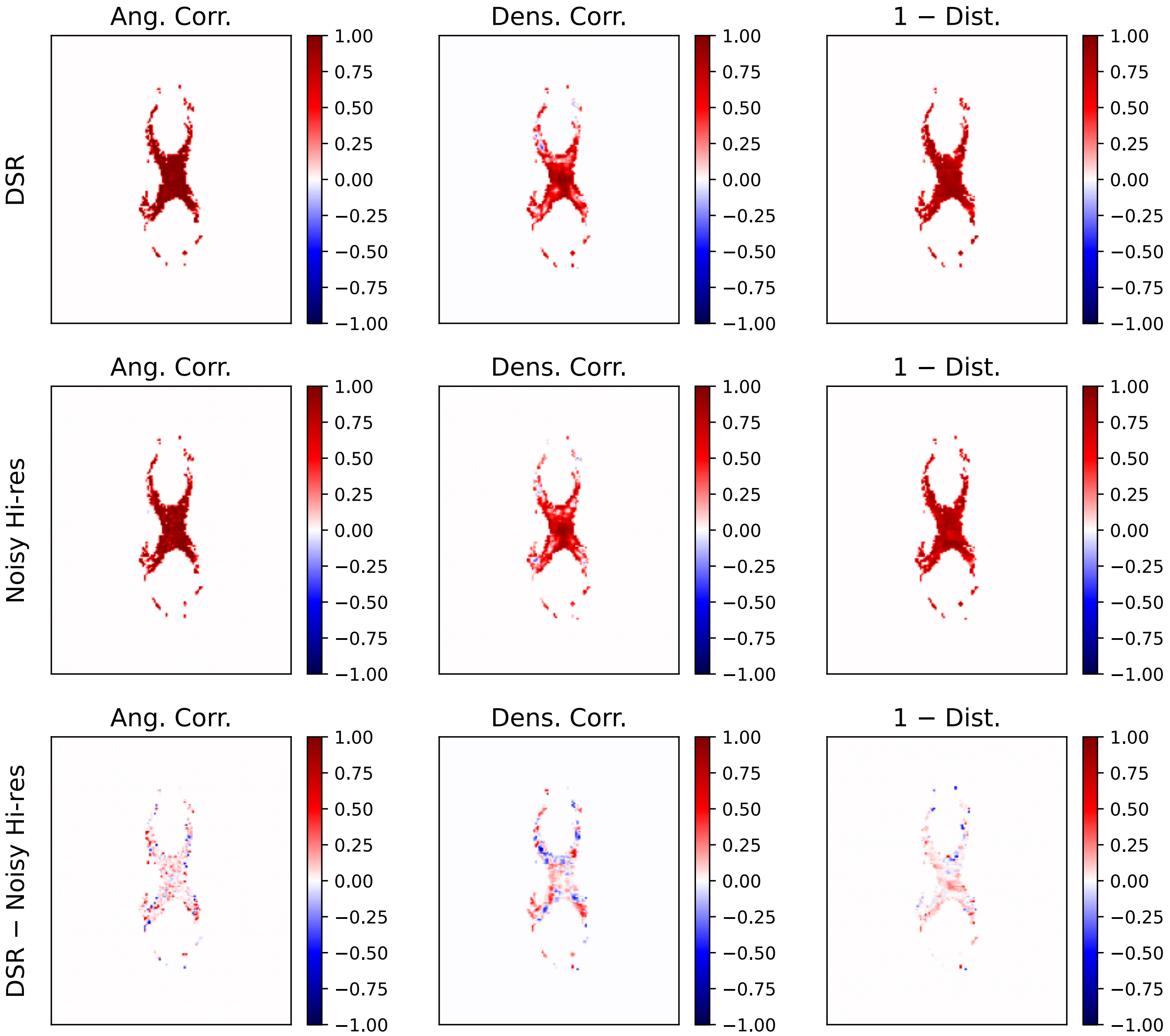

Supplementary Figure 6. Fidelity metrics maps for tracts obtained by seeding voxels only belonging to the corpus callosum (CC). From left to right: angular correlation (the higher the better); density correlation (the higher the better); and 1− Distance between streamlines (the higher the better). From top to bottom: maps obtained by comparing the ground truth with data upsampled via DSR; maps obtained by comparing the ground truth with the noisy high-resolution data; and the difference between the previous two maps.

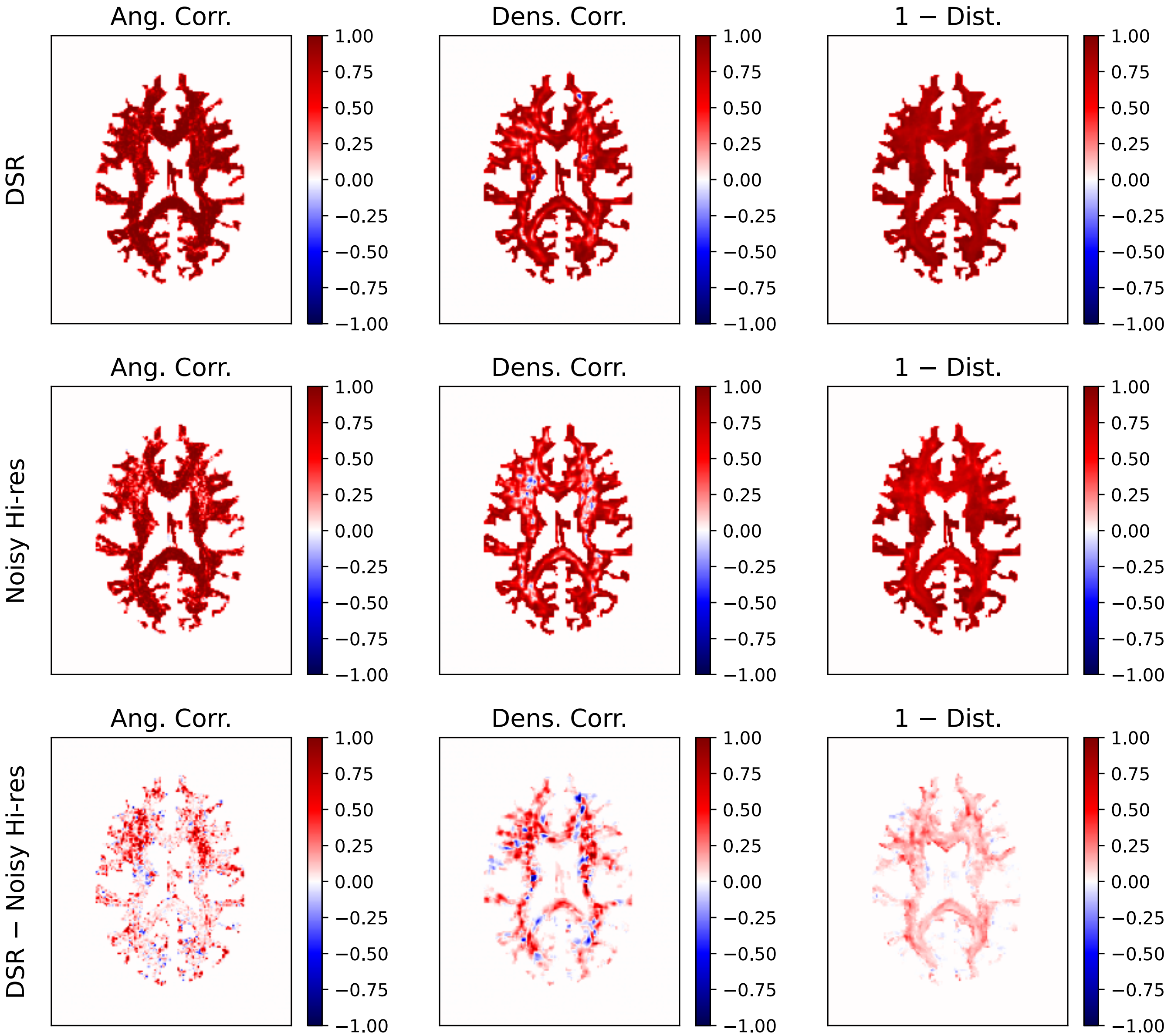

Supplementary Figure 7. Fidelity metrics maps for tracts obtained by seeding all voxels only belonging to white matter (WM). From left to right: angular correlation (the higher the better); density correlation (the higher the better); and 1− Distance between streamlines (the higher the better). From top to bottom: maps obtained by comparing the ground truth with data upsampled via DSR; maps obtained by comparing the ground truth with the noisy high-resolution data; and the difference between the previous two maps.

are 3-dimensional vectors with their corresponding amplitude (or weight). For these two sets of vectors, referred to as the target distribution, U, and the estimated one V, the EMD method aims to minimize the required work needed for the estimated set to match the target one. The operation which defines the work is:

$$W(\mathrm{U},\mathrm{V}) = \min_F \sum_{i=1}^{m} \sum_{j=1}^{n} f_{i,j}\, d_{i,j}\,, \tag{2}$$

where F represents the optimal flow variables $f_{i,j}$ required so that the estimated set of vectors V matches the target one U, and $d_{i,j}$ is the distance between the vectors $\boldsymbol{u}_i$ and $\boldsymbol{v}_j$. The distance matrix employed is the angle between the compared vectors, i.e.,

$$d_{i,j} = \arccos\left(\left|\hat{\boldsymbol{u}}_i \cdot \hat{\boldsymbol{v}}_j\right|\right)\,, \tag{3}$$

where $\hat{\boldsymbol{u}}_i$ and $\hat{\boldsymbol{v}}_j$ are the vectors $\boldsymbol{u}_i$ and $\boldsymbol{v}_j$ normalized to unit length, so that each d_i, j takes values between 0° and 90°. Equation 2 is solved under the additional constraints:

$$\sum_{j=1}^{n} f_{i,j} = w_i\,, \tag{4a}$$

$$\sum_{i=1}^{m} f_{i,j} = p_j\,, \tag{4b}$$

$$f_{i,j} \geq 0, \tag{4c}$$

where w_i and p_j are the weights associated with each vector in U and V, respectively. Also note that $\sum_{i=1}^{m} w_i = \sum_{j=1}^{n} p_j = 1$, since the main fODF peak target and estimated distributions are fully defined by m and n peaks, respectively. Essentially, these constraints ensure that the initial vector distribution V is transformed to the target vector distribution U. Equations 2 and 4 can then be solved using linear programming. We employed the `optimize.linprog` function of the `scipy` package in Python. Finally, the similarity metric can be obtained by solving Equation 2 with the optimal flow F, yielding:

$$\theta = \sum_{i=1}^{m} \sum_{j=1}^{n} f_{i,j}\, d_{i,j}\,, \tag{5}$$

where θ is the the total work needed for the set of vectors V to be transformed to vectors U. Since the distance metric employed in this implementation was the angle between vectors, θ can also be interpreted as the angular error between vectors, where a minimum error of 0° means both sets of vectors are exactly the same. The larger the angular error, the less similar these set of vectors are.
